# The influence of James and Darwin on Cajal and his research into the neuron theory and evolution of the nervous system

**DOI:** 10.3389/fnana.2014.00001

**Published:** 2014-01-29

**Authors:** Francisco R. M. Ferreira, Maria I. Nogueira, Javier DeFelipe

**Affiliations:** ^1^Laboratory of Neuroscience, Institute of Psychology, University of São PauloSão Paulo, Brazil; ^2^Laboratory of Neuroscience, Institute of Biomedical Sciences, University of São PauloSão Paulo, Brazil; ^3^Laboratorio Cajal de Circuitos Corticales, Centro de Tecnología Biomédica, Universidad Politécnica de Madrid e Instituto Cajal, MadridSpain; ^4^Centro de Investigación Biomédica en Red sobre Enfermedades NeurodegenerativasMadrid, Spain

**Keywords:** neural plasticity, evolution of the nervous system, reticular theory, neuron theory, history of neuroscience

## Abstract

In this article we discuss the influence of William James and Charles Darwin on the thoughts of Santiago Ramón y Cajal concerning the structure, plasticity, and evolution of the nervous system at the cellular level. Here we develop Cajal’s notion that neuronal theory is a necessary condition to explain the plasticity of neural connections. Although the roots of the term “plasticity” in reference to neuroscience are not completely clear, Cajal was an important figure in the propagation and popularization of its use. It is true that he carried out a large *number* of studies throughout his career in favor of the neuronal theory, but perhaps one of the most interesting aspects of his studies was his innovative capacity to interpret structure as being the result of evolutionary mechanisms, i.e., natural selection. This capacity would ultimately lead Cajal to the conclusion that, in relation to the histology of the nervous system, such selection occurs in the establishment of connections between cells. The present article is divided into five sections: (1) Learning and general notions of organic plasticity in the 19th century; (2) The idea of “mental” plasticity proposed by James; (3) Neuronal theory and “structural” plasticity: general considerations; (4) Evolutionary factors of the nervous system in Cajal’s work; and (5) Final considerations.

## INTRODUCTION

The origins of modern neuroscience was highly influenced by the revolutionary biological thinking that arose in the second half of the 19th century with the theory of Charles Darwin (1809–1882) concerning the evolution of the species through natural selection. An important example is the strong influence of the theory of evolution on the embryologists (Coleman, 1983). In this early period, explanations were based on the phylogenetic evolution of organisms. The relationships between the development of the organism (ontogenesis) and of the species (phylogenesis) resulted in the theory of recapitulation, which proposed that in developing from embryo to adult, animals go through stages resembling or representing successive stages in the evolution of their remote ancestors. This idea influenced naturalistic thinking (Gould, 2010):

“*Naturalists had at the time an infinite belief in the actual existence of a straight parallel and even may be an identity between the developments of a full-grown chick from a chicken egg, for instance, and the entire species of chicks from a more primitive bird.” *

(Coleman, 1983, p. 85)

The overall way of thinking of academic circles in Europe in the second half of the 19th century was influenced by the theories of evolution (Baumer, 1977), with the reach of this influence extending beyond the boundaries of biology and similar sciences.

As a result, given the general importance placed on evolution by the academic community at the time, it follows that the study of the nervous system would be approached from an evolutionary standpoint. This paper aims to examine, at the cellular level, the notion of evolution of the nervous system presented by Santiago Ramón y Cajal (1852–1934). The notion that neuronal theory is a necessary condition to explain the plasticity of neural connections will also be explored. It is important to highlight that the studies in the field of neuroscience converged increasingly in the twentieth century to the point that the notion of the plastic nervous system was generally accepted as a critical mechanism for learning and memory (Barlow, 1972, 1995; Azmitia, 2002, 2007; Fields and Stevens-Graham, 2002; DeFelipe, 2006).

The sheer number of studies in favor of the neuronal theory that Cajal carried out over the course of his career was impressive. However, perhaps one of the most interesting aspects of his studies was his innovative capacity to interpret the structure as being the result of evolutionary mechanisms, i.e., natural selection and his proposal that, ultimately, in relation to the histology of the nervous system, such selection might occur in the establishment of connections between cells.

The present article has been divided into five sections: (1) Learning and general notions of organic plasticity in the 19th century; (2) The idea of “mental” plasticity proposed by William James (1842–1910); (3) Neuronal theory and “structural” plasticity: general considerations; (4) Evolutionary factors of the nervous system in Cajal’s work; (5) Final considerations.

## LEARNING AND GENERAL NOTIONS OF ORGANIC PLASTICITY IN THE 19TH CENTURY

In the world of neuroscience the notion of learning encompasses a wide range of events involving both biological factors and those related to the interaction between the organism and its environment at both, a biological dimension and the role of interaction between the organism and its environment. Learning is closely associated with memory mechanisms and implies plastic changes in the brain at different levels (genes, molecules, synapses, etc.), which lead to structural and functional reorganization of neural networks.

Here, we will not attempt to perform an exhaustive review of the concept of learning, but rather our intention is to briefly outline this area in order to proceed with a historical and conceptual analysis of the notion of plasticity in biological terms, since learning and plasticity are closely related.

The concept of plasticity as an indicator of changes in the organism, at both the anatomic and behavioral level, was extensively discussed within the area of psychology in the late 19th century and early 20th centuries. Analysis of the relationships between the biological and the psychological traits of the organism was present in scientific debates around this time period (Galton, 1880; Ebbinghaus, 1885; Calkins, 1896; Thorndike, 1898; Small, 1901; Yerkes, 1901; Pavlov, 1904; Köhler, 1917).

It should be noted that the experimental approach for the study of the psychology of learning attracted considerable interest from psychologists at the time. It was then that experiments with labyrinths were first used to investigate the cognitive and emotional skills of animals (Small, 1901; Yerkes, 1901). The theory guiding most of these studies assumed that our mind operates by association (the association theory) of elements subject to experimental treatment due to the relation between stimulus and response.

In a landmark study, the sociologist and politician Leonard T. Hobhouse (1864–1929), opposed the notion of learning as a mere process of fixing connections and associations between stimulus and response (Hobhouse, 1901). Such new notion became widespread thanks to a study by Wolfgang Köhler (1887–1967) using chimpanzees (Köhler, 1917). Köhler concluded that chimpanzees can learn by associating stimuli and not only through the stimulus-response relation.

It was James (1879), first drew attention to the idea that there were anatomical changes associated with plasticity although objective results were not provided to corroborate this idea. In the next section, we will address James’ notion of plasticity and then discuss how Cajal presents this notion in relation to the connections between the cells of the nervous system, which in our opinion is a broader notion with a better set of experimental results compared to other contemporary notions.

## THE IDEA OF PLASTICITY PROPOSED BY WILLIAM JAMES

William James is considered a pioneer in transforming psychology into an independent science (Kinouchi, 2009). It took 12 years to write the well-known reference book for beginners in psychology: *The Principles of Psychology* (James, 1890). For the purposes of our analysis, we have examined two chapters in detail, namely chapter IV – *Habit* – and chapter V – *The automaton theory*. Both chapters were published before the book. Chapter IV was originally published in February 1887 in *Popular Science Monthly* under the title *The laws of habit* and chapter V was originally published in 1879 in *Mind* # 4 (pp. 1–22). However, in the book *The Principles of Psychology *(James, 1890), these chapters were revised and updated based on the debate established. Thus, we will refer to the book instead of the original chapters.

In the first chapter of the book *The Principles of Psychology*, James (1890) establishes as the central theme what he considers to be the subject matter of psychology, as per the quote below.

*“(…) the fact that the brain is the one immediate bodily condition of the mental operations is indeed so universally admitted nowadays that I need spend no more time in illustrating it, but will simply postulate it and pass on. The whole remainder of the book will be more or less of a proof that the postulate was correct.”*

(James, 1945, p. 16)

According to James, there was a hierarchical division of the nervous system in such a way that the lower centers respond to sensory stimulus, while the cerebral hemispheres are responsible for perception and conscious actions. Perceptions involve the grouping of sensations while conscious considerations are expectations of sensations to be felt based on previous experience of the sensations felt. In this explanatory model, memory has a central role. James indicates that our memory is located in the cerebral hemispheres. He also affirms that cerebral functions represent the crucial differences in terms of the variety of responses seen between an animal that has no cerebral hemispheres and another animal with cerebral hemispheres. While the latter would respond to absent objects the former would only respond to present stimuli.

In the section where he writes about training the cerebral hemispheres, James justifies his idea that reflexes may be influenced by both physical and psychic factors:

*“I hope that the reader will take no umbrage at my so mixing the physical and mental, and talking of reflex acts and hemispheres and reminiscences in the same breath, as if they were homogeneous quantities and factors of one causal chain. I have done so deliberately; for although I admit that from the radically physical point of view it is easy to conceive of the chain of events amongst the cells and fibers as complete in itself, and that whilst so conceiving it one need make no mention of ideas, I yet suspect that point of view of being an unreal abstraction. Reflexes in centers may take place even where accompanying feelings or ideas guide them.”*

(James, 1945, p. 33)

The question underlying such an argument is how hemispheric processes that correspond to what James called recollection of the “spirit” (mental processes) can be organized. In answering this question, it is important to consider, firstly, the process that occurs in the brain when it is stimulated. For instance, the visual perception of an object will be reproduced giving an idea of the same object when internally stimulated by other brain processes. Another clue is that when processes are stimulated jointly or immediately after one another, a stimulus arising from any of the given processes tends to stimulate the other processes in a sequential order. James named this second hypothesis the law of association. The third hypothesis states that any stimulus spreading to the lower centers tends to also spread to the higher centers (cerebral cortex) and produce a general idea. The result of these three hypotheses is that all ideas tend to ultimately produce or restrain a motor response that would otherwise be produced.

We notice that James refers to a problem that was the focus of academic debate in the second half of the 19th century, namely expanding physiological explanations to account for thoughts and ideas. James’ line of thinking does not align him with either side of the debate. He found in Darwinian Theory a way to transcend these two sides between physiological determinism and social determinism (Kinouchi, 2006).

A crucial moment for this discussion took place in the last three decades of the 19th century. The 1870s were the beginning of a period that basically rejected the phrenological view that discrete cortical areas represented individual functions. An alternative to this idea was that although the cerebral hemispheres were associated with certain functions, they were not acting in isolation, but rather in conjunction with the entire organism. Thus, this idea of continuity or cooperation between the parts was not incompatible with the existence of reflex, such as motor reflexes associated with the spinal cord. This scenario led researchers to question whether it was the cerebral cortex alone that creates states of consciousness. With this idea of cooperation in mind, perhaps it would not be unreasonable to suggest that there are also levels of consciousness in the lower centers? This and other questions arose in studies carried out during this period

Another important concept is the notion of habit. James distinguished inherent or instinctive habits from variable or acquired habits – with this second category of human beings’ habit being acquired by education or learning.

James begins his considerations on the changes that occur in organisms by analyzing the physical world. The particles in the physical world do not change due to their nature. However, mass that is made up of matter can have changes. Such changes occur in a compound structure. James wrote:

“(…) Plasticity, then, in the wide sense of the word, means the possession of a structure weak enough to yield to an influence, but strong enough not to yield all at once. Each relatively stable phase of equilibrium in such a structure is marked by what we may call a new set of habits. Organic matter, especially nervous tissue, seems endowed with a very extraordinary degree of plasticity of this sort; so that we may without hesitation lay down as our first proposition the following, that the phenomena of habit in living beings are due to the plasticity of the organic materials of which their bodies are composed.”

(James, 1945, p. 106)

Therefore, the starting point for the notion of plasticity explored by James was a certain property of the physical world, with organic matter being the ultimate product. James was optimistic about the likelihood of future explanations accounting for changes to the most intimate part of organic matter.

The early ideas on the process of forming a habit can be better understood in two essays from the 1870s, one by Léon Dumont (1837–1877) and the other by Albert Lemoine (1824–1874; Dumont, 1876; Lemoine, 1876). James appears to have been influenced by Dumont’s essay on the physical characteristics of habit formation.

James also interpreted the adaptive properties of consciousness as being a plastic mechanism. In the chapter on The Automaton-Theory, James presents a widespread theory in the second half of the 19th century, in which the notion of the “reflex” in the nervous system (a behavior that is mediated via the reflex arc) was extrapolated to conscious acts to explain conscious behavior. Based on this concept, attributing a function to consciousness in mechanical terms is not necessary, since this theory explains the causal relations between stimulus and motor response without the necessity for an external agent whose nature differs from that of the organic elements involved.

According to Kinouchi (2006), James does not fully agree with this physiological view alone. The idea of consciousness in terms of evolution has a unique role in James’ line of thinking. Indeed, evolution would to an extent explain the role of consciousness. What would be the possible deficiencies of the nervous system in animals whose consciousness appear to be more developed? In James’ view, the key characteristic that indicates such possible deficiencies is instability.

This brief explanation of the idea of plasticity from James’ perspective leads to some key points. Firstly, plasticity occurs in the “matter” of the nervous system. Nevertheless, James does not provide experimental results to corroborate this idea. Another key point refers to the adaptive role that consciousness has by providing organisms with the capacity to change in response to the environment. In the next section, we will analyze how Cajal introduced the concept of plasticity at the level of connections between the cells of the nervous system.

## NEURONAL THEORY: GENERAL CONSIDERATIONS

In the 19th century there was a widespread idea that nerve cells linked to each other through anastomosis (fusion), similar to pieces of a tubing system.

At that time, several authors supported the reticular theory proposing different types of networks, including Albrecht Von Kölliker (1817–1905), a major scholar in anatomy and embryology (Kölliker, 1868). However, it was Joseph von Gerlach (1820–1896), an enthusiast of Kölliker’s ideas on the fusion of the nervous system, who really developed the reticular theory (Gerlach, 1872) through the use of a procedure of staining with ammoniated carmine and gold chloride that he introduced. He observed a very fine reticulum of nerve cell processes in the gray matter of the cerebral cortex, cerebellar cortex, and spinal cord and such observations led him to propose that the nerve impulse travels from cell to cell through fiber networks formed as meshes. Accordingly, nervous tissue would consist of a reticulum comprising a large number of pieces that were physically interconnected (Shepherd, 1991; Jones, 1994).

In the mid 1860s – a period in which the scientific community leaned toward the reticular theory – the ideas of Kölliker and Gerlach, who were experts in the subject, were readily accepted since there was no empirical evidence that ruled out this theory. This situation would start to change with a new method for staining tissue, the black reaction (*la reazione nera*), a method developed by Camillo Golgi (1843–1926) in 1873.

**FIGURE 1:**
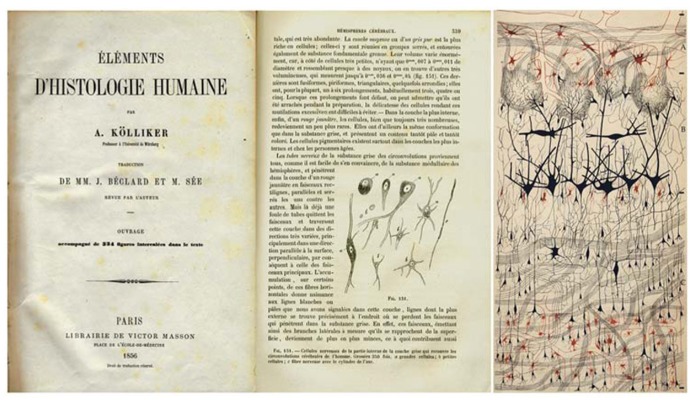
**Illustrations showing the evolution of the visualization of the structure of the nervous system.** Left: drawing by Kölliker to show different cell types in the cerebral cortex (Published in 1856 in the French edition of Kölliker (1852), classic book Handbuch der Gewebelehre des Menschen). Right: the first illustration of a histological preparation by Golgi with his silver nitrate method (“reazione nera”; black reaction) to study the olfactory bulb of the dog (Golgi, 1875). Taken from DeFelipe (2010a).

Thanks to the Golgi method, it was possible for the first time to observe neurons and glia in a histological preparation with all their parts (cell body, dendrites, and axon, in the case of neurons; cell body and processes in the case of glia (**Figure 1**; see below).

Cajal used this revolutionary technique immediately after the psychiatrist and neurologist Luis Simarro (1851–1921) showed Cajal in 1887 a Golgi-impregnated preparation in his private laboratory. Cajal started to use the technique and went on to analyze practically the entire nervous system in several species. Cajal (1888) had published his first important article based on results obtained with this method in the avian cerebellum. In this study entitled *Estructura de los Centros Nerviosos de las Aves*, Cajal confirmed Golgi’s conclusion that dendrites end freely but, unlike Golgi, Cajal added the decisive conclusion that this also applies to axons and their branches:

“We have carried out detailed studies to investigate the course and connections of the nerve fibres in the cerebral and cerebellar convolutions of the human, monkey, dog, etc. We have not been able to see an anastomosis between the ramifications of two different nervous prolongations, nor between the filaments emanating from the same expansion of Deiters [axons]. While the fibres are interlaced in a very complicated manner, engendering an intricate and dense plexus, they never form a net […] it could be said that each [nerve cell] is an absolutely autonomous physiological canton [unit].“

(Santiago Ramón y Cajal, 1888)

Thus, by using this technique Cajal came to a conclusion that differs greatly from that defended by the followers of the reticular theory. He suggested that, instead of forming a continuous network, single nerve cells communicate with one another through a specific mechanism by contact or synapse, although the term “synapse” was coined later by Charles Sherrington (1857–1952) in 1897 to describe the hypothetical one-way contact between axon terminals and somata or dendrites (Foster and Sherrington, 1897).

**FIGURE 2:**
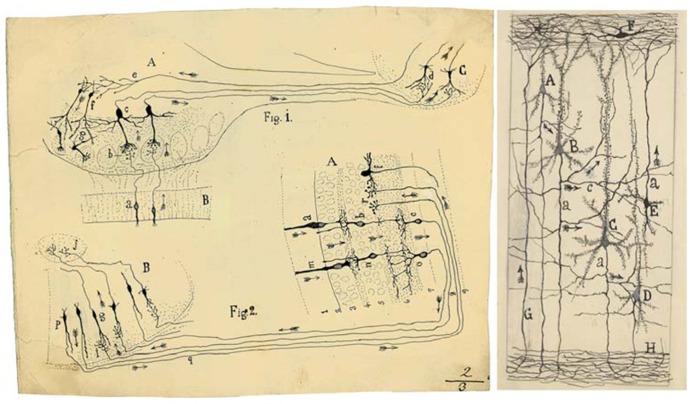
**(Left) Cajal’s scheme showing the flow of current (arrows) in the visual and olfactory systems to support the Law of Dynamic Polarization.** This drawing was reproduced in his article *Significación fisiológica de las expansiones protoplásmicas y nerviosas de las células de la substancia gris* (Rev. Ciencias Méd., 22: 673–679; 715–723, 1891). *Fig. 1.* Scheme of cellular connections in the olfactory mucosa, olfactory bulb, tractus, and olfactory lobe of the cerebrum. The arrows indicate the direction of the currents. *A*, olfactory bulb; *B*, mucosa; *C*, olfactory lobe. *a, b, c, d*. One-way or centripetal pathway through which sensory or olfactory excitation passes. *e, f, g,* Centrifugal pathway through which the [nervous] centres can act on the elements of the bulb, granules and nerve cells, whose protoplasmic processes penetrate the glomeruli. *Fig. 2.* Scheme of the visual excitation pathway through the retina, optic nerve and optic lobe of the birds. *A*, retina; *B*, optic lobe. *a, b, c,* represent a cone, a bipolar cell and a ganglion cell of the retina, respectively, the order through which visual excitation travels. *m, n, o,* parallel current emanating from the rod also involves bipolar and ganglion cells. *g*, cells of the optic lobe that receive the visual excitation and transfer it to *j*, the central ganglion. *p, q, r*, centrifugal currents that start in certain fusiform cells of the optic lobe and terminate in *r*, in the retina at the level of the spongioblasts; *f*, a spongioblast. (Right) Schematic drawing by Cajal to show synaptic connections and the possible flow of information through neural circuits in the cerebral cortex. Taken from *Neuronismo o reticularismo? *(Cajal, 1933). *A*, small pyramid; *B* and *C*, medium and giant pyramids respectively; a, axon; [*c*], nervous collaterals that appear to cross and touch the dendrites and the trunks [apical dendrites] of the pyramids; *H*, white matter; [*E*, Martinotti cell with ascending axon]; *F*, special cells of the first layer of cerebral cortex; *G*, fibre coming from the white matter. The arrows mark the supposed direction of the nervous current.

Cajal proposed that neurons could be divided into three functionally distinct regions: a receptor apparatus (formed by the dendrites and soma), an emission apparatus (the axon) and a distribution apparatus (terminal axonal arborization). Thus, the new ideas about the connections between neurons led to novel theories concerning the relationship between neuronal circuits and brain function (DeFelipe, 2010b) and it was possible to trace the first circuit diagrams of the brain (**Figure 2**).

An important consequence of the Neuron Theory was the introduction of the concept of plasticity based on changes on the structure of the nervous system. Cajal had indeed used the term plasticity in the transactions of the International Medical Congress held in Rome in 1894 published in *La Veterinaria Española* (Cajal, 1894). Cajal explained his theory about cerebral gymnastics, clearly stating that the capacity to increase neuronal connections was a plastic mechanism in response to a continued stimulus.

“*As opposed to the reticular theory, the theory of the free arborization of the cellular processes that are capable of developing seems not only the most likely, but also the most encouraging. A continuous pre-established net – like a lattice of telegraphic wires in which no new stations or new lines can be created – somehow rigid, immutable, incapable of being modified, goes against the concept that we all hold of the organ of thought that within certain limits, it is malleable and capable of being perfected by means of well-directed mental gymnastics, above all during its period of development. If we did not fear making excessive comparisons, we would defend our idea by saying that the cerebral cortex is similar to a garden filled with innumerable trees, the pyramidal cells, which can multiply their branches thanks to intelligent cultivation, sending their roots deeper and producing more exquisite flowers and fruits every day*.”

In this publication, Cajal applied the words “dynamism,” “force of internal differentiation,” “adaptations (of the neurons) to the conditions of the environment” and “plasticity,” among others, to describe the potential of the brain to adapt to the environment. Cajal had been invited to deliver a plenary lecture at this congress and, although he could not attend, it is likely that it was there that the term “plasticity” became popular (DeFelipe, 2006).

There is no doubt that some of Cajal’s ideas regarding the influence of the environment, such as the influence of education in mental activities, had been proposed by a number of physicians, teachers and philosophers long before. Indeed, Sigmund Freud (1856–1939) used the word plasticity before Cajal, as did other neurologists and psychiatrists of the time, when referring to the “plasticity of psychic material,” inferring that the brain or nervous system as a whole is “plastic.” As discussed above, in *The Principles of Psychology* (James, 1890), the term “plasticity” referring to the nervous system appears in several passages, particularly to explain habits. James (1890) uses “plasticity” in a broad sense that does not necessarily imply a change in the external form of the structure, but may be “invisible and molecular, as when an iron rod becomes magnetic.” Nevertheless, Cajal’s contribution was crucial in trying to explain these facts from a structural or connectional point of view based on the Neuron Theory (DeFelipe, 2006).

## EVOLUTIONARY ASPECTS OF THE NERVOUS SYSTEM IN CAJAL’S WORK

This topic is mainly based on the first five chapters of Cajal’s classic book: *Textura del sistema nervioso del hombre y de los vertebrados* (Cajal, 1899–1904). Readers who are interested in going into further detail on this subject should consult Swanson (2007) and the references contained therein.

The idea of the nervous system being the central organism in the process of organizing and creating behaviors is the basis for most of the histological studies on the fine structure of the nervous system that approach this topic from an evolutionary perspective-properties such as sensation, thinking and willpower – when considered exclusively from an evolutionary point of view – are all a result of the evolution of the nervous system. Irritability was considered to be a fundamental property of the cell in the second half of the 19th century (e.g., Maestre de San Juan, 1885). With regards to this, the presence of flagella in certain locations in infusorians (nowdays named Cnidarians) led to a greater range of motor and sensory possibilities, which in turn translated into development beyond the level of the organism itself, that is, development at the evolutionary level (Cajal, 1899–1904).

The organization of sensory phenomena and the “division of work” will only occur, according to Cajal, in pluricellular organisms. Cajal frequently uses the expression “functional solidarity” to describe the functional specificity of organisms. He defines what we can call the origin of a proto-nervous system in coelenterates and mentions the work of zoologists Blanchart, Hertwit, Zoja, and Wolff, who identified in polyps a “nervous system” comprising two distinct classes of neurons: motor neurons and sensory neurons.

**FIGURE 3:**
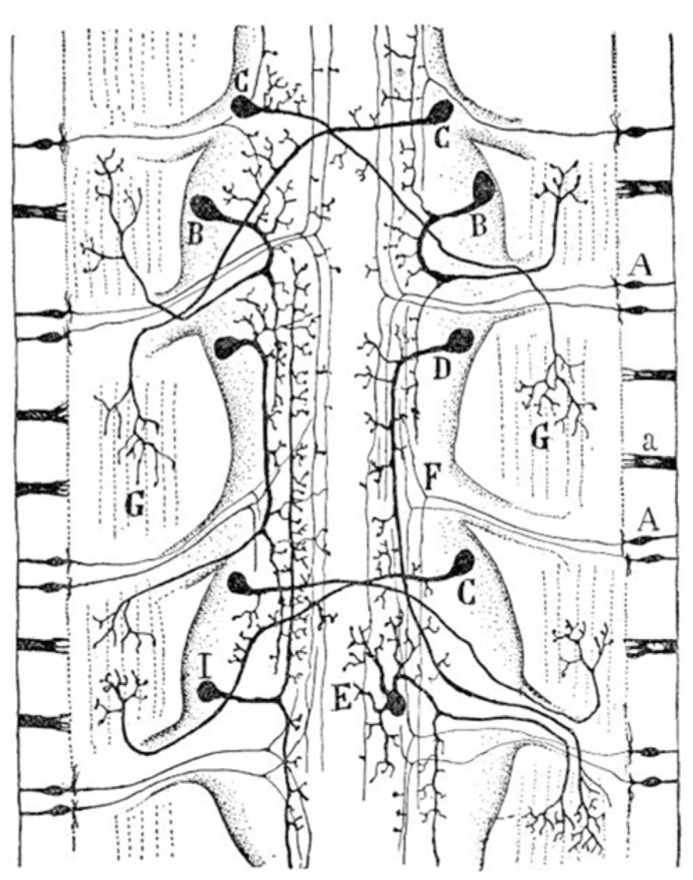
**Diagram of the sensory and motor nervous system of a worm (composite of two figures, one from Retzius and another by v. Lenhossék).** **(A)** Sensory cells of the skin; **(B)** ipsilateral motor cells of central ganglia; **(C)** crossed motor cells; **(D)** ipsilateral longitudinal motor cells; **(E)** multipolar motor cell; **(G)** terminal ramifications of motor neurons in muscles; **(I)** interganglionar association cells. Taken from Cajal (1899–1904).

A third class of nerve cells appear in worms (**Figure 3**); the association neuron (interneuron). Cajal believe that understanding these first agglomerates of nerve cells (proto-nervous system) was of utmost importance to understand the origin and differentiation of the nervous system. The following quotation by Cajal identifies the advantages that organisms gained after the differentiation of the association neuron:

“*… sensory excitation can propagate not only to the motor cells of a particular ganglion but also to those residing in other ganglia; *in this way, the animal is *capable of reacting *after being stimulated at any point on the skin, *triggering most or perhaps the totality of the locomotor apparatus.”*

(Cajal, 1899–1904, p. 04)

Cajal further describes the appearence of a fourth type of nerve cell, the psychomotor neuron, which is located in the cerebral ganglion or animal brain, from where it controls the others cells. Cajal believed that the information that reaches the skin activates the bipolar sensory cell and travels to the corresponding ganglion center, where the connection between the central arborization and the outgoing motor neurons facilitates the innervation of the muscle to be triggered. When considering animals that are more complex, Cajal affirms that a new element of connection – the association neuron – appears between the sensory neuron and the motor neuron.

The psychomotor cell found in the cerebroid ganglion of invertebrates and in the brain of vertebrates directs information from nervous centers (via voluntary “orders”) and stimulates motor neurons. Cajal proposes a classification of the zoological scale in terms of the evolutionary order of appearance of cell types: (1) unicells and sponge-like structures: era of irritability; (2) celenterates: era of fundamental neurons; (3) lower invertebrates: era of association neurons; (4) vertebrates: era of the psychomotor neurons.

The above classification proposed by Cajal does not assume the existence of leaps among the mentioned groups of animals. In terms of evolution, the perfecting that each era imprints on the prior era directs the psychomotor neurons toward what Cajal called “functional solidarity” of the entire organism:

“*The preponderance and directing excitatory or inhibitory action of the cerebroid ganglion is one of the most surprising phenomena provided by the evolution of the nervous system. [This phenomenon] leads to the emergence of memory, will and intelligence. Since there are no significant structural, morphological, chemical and evolutionary differences between neurons of the cerebroid ganglion and those populating the esophageal and abdominal ganglia, what is the reason for this hierarchical superiority reached by the encephalic ganglion?*”

(Cajal, 1899–1904, p. 06)

The higher complexity of the psychomotor neuron is related to how information from the environment is processed. Cajal believes the main cause of this phenomenon is the existing dynamic relations established between the cerebroid ganglion and the outside world. Rather than receiving from the latter mere tactile and thermal stimulation – Like the abdominal ganglia, the cerebroid ganglion receives from sensory organs impressions which had already been organized, rather than mere tactile and thermal stimulation. These impressions include real images of the outside world that have fixed relations in time and space – the cerebroid ganglion receives from sensory organs impressions which have already been previously organized – real images of the outside world that have fixed relations in time and space.

In the preface of the book by Pedro López-Peláez, *Anatomía normal de la médula espinal humana* (Cajal, 1897), Cajal described how Corti’s organ for hearing and cones and rods of the retina act as filters for complex patterns of waves received from the environment by selecting and organizing them in sound and image, respectively, and subsequently projecting them to the cerebral cortex, which transforms them into sensations, ideas and volitions. In the words of Cajal:

“*The cerebrum of the vertebrates or the encephalic ganglion of invertebrates need not create images; they are given to them perfectly organized by the sense organs, with intensities proportional to the energy of the stimuli, which marvelous architecture constitutes the primordial cause of the superior mental activity of animals. In a word, the morphology and chemical composition of a cell, although very important for the type of psychic operation, do not exclusively determine the hierarchy of this operation, which chiefly depends on the quality of the excitation received from the outside world.”*

(Cajal, 1899–1904, pp. 06–07)

Cajal agreed with the explanation given by Joseph Pierre Durand (1826–1900) and Auguste Henri Forel (1848–1931) on the conscious response from the spinal cord (Forel, 1896; Durand quoted in Cajal, 1899). If this relationship (stimuli generated from the environment) is unclear and diffused, i.e., if there is no precision in the relationship between extension and form, the raw material of sensation actually triggers motor impulses and conscious representations together with the basic response from the spinal cord. Cajal gives the example of tactile and thermal information that reaches the abdominal ganglion of invertebrates and the spinal cord of vertebrates.

The hypothesis of attributing consciousness to lower nervous centers, in particular to the spinal cord, was initially defended by Eduard Friedrich W. Pflüger (1829–1910) and subsequently expanded by Joseph Pierre Durand (1826–1900) and Auguste Henri Forel (1848–1931). This objective hypothesis tried to bridge the gap left by the differences between the cerebral ganglion and the sympathetic and spinal cord nervous centers. Cajal believed that if the optic nerve ended directly in the spinal cord, then the spinal cord would create not motor stimuli but visual images. Building upon this idea, Cajal mentions the principle of Pflüger which assumes that “the cause of an organic necessity is also the cause of satisfying this necessity.”

The considerations presented so far in the evolutionary concept of major cell types of the nervous system in the zoological series aim to demonstrate the thesis defended by Cajal in which he affirms that the differentiation of cerebroid operations is subject to special sensory relationships. A widespread thesis on the matter was proposed by Theodor Meynert (1833–1892), (Cajal, 1899–1904). In Meynert’s view, the functional diversity of nerve cells was related to the differences in their peripheral connections, and his intention was to explain why different functional regions of the cerebral cortex perform such different activities despite the fact that the structure of these regions appears to be identical.

The solution to this problem lies in the explanation of why parts of the epidermis that are metamerically associated with the cerebral ganglion become differentiated to form an eye. Herbert Spencer (1820–1903) explains that the appearance of sensory organs or structures results from combined operations between adaptation and natural selection (Spencer, 1871). This explanation influenced Cajal greatly, as did Spencer’s line of thinking as a whole. However, Cajal believed that it is not easy to fully account for this problem via a progressively evolutionary approach. We note below the argument defended by Cajal:

*“We must confess that, even applying the principle of natural selection, it is impossible to explain satisfactorily these marvelous apparatuses of relationship [with the environment] which are, as we have said, the probable efficient cause of the superior dynamic hierarchy of the cephaloid ganglion and of the directing role that it exerts over all other ganglion foci.”*

(Cajal, 1899–1904, p. 08)

Cajal proceeds with his line of reasoning regarding the difficulty in satisfactorily explaining the leap from a sensory mechanism to a more developed level by considering the progression from one organism to another during evolution. The example given by Cajal is the panoramic vision of fish, reptiles and amphibians – animals in which the optic nerve fibers cross over completely. In higher mammals, vision is binocular and within a single field. The optic nerve in these animals crosses only partially, with part of it remaining uncrossed. Cajal refers to the fact that this partial arrangement can result in diplopia, causing imperfect vision when compared to lower vertebrates. Although highlighting this and other obstacles to the idea of progressive evolution, Cajal affirms in the French version of the *Textura* (Vol. 1, p. 10):

“*This and other arguments do not lead us to reject the principle of selection. We have herein advanced this argument to show the need to accept that, concerning progressive evolution, there are factors that are as yet unknown*.”

Further improvement to the nervous system highlighted by Cajal was related to the significant levels of development achieved by sensory organs and that they are distinct in vertebrates, especially in mammals, as segments of various structures, such as the forebrain midbrain, intermediate brain and hindbrain. In relation to invertebrates, Cajal mentions the double ganglionic chain that they have, which blends into a single nerve cord.

In order to automatically control the background vegetative processes of the organism (such as digestion, circulation, secretion, etc.), Cajal states that there was the differentiation of a new ganglionic chain, the sympathetic ganglion, whose functions are partially independent from the cerebrospinal system.

Finally, a favorite subject of research for Cajal was the question of what is special about the neocortex of humans and how does it differ from that of other species? In the words of Cajal:

“*At that time, the generally accepted idea that the differences between the brain of [non-human] mammals (cat, dog, monkey, etc.) and that of man are only quantitative, seemed to me unlikely and even a little offensive to human dignity … language, the capability of abstraction, the ability to create concepts and finally, the art of inventing ingenious instruments … do [these facets] not seem to indicate (even admitting fundamental structural correspondences with the animals) the existence of original resources, of something qualitatively new which justifies the psychological nobility of Homo sapiens? Microscope at the ready, I then launched with my usual ardor to conquer the supposed anatomical characteristic of the king of Creation, to reveal these enigmatic strictly human neurons upon which our zoological superiority is founded.”*

(Cajal, 1917)

Thanks to the discovery of the Golgi method, it was possible to start the detailed study of the nervous system in order to compare the neuronal organization between different brain regions within a given species and between species. The idea was to determine whether it was possible to explain functional specialization through structural specialization:

“… for example, if an organizational detail is found exclusively in or is particularly exaggerated in the visual cortex, we will be justified in suspecting that it has something to do with [cerebral visual function]. Conversely, if an anatomical detail is repeated equally in all cortical regions, we will be justified in assuming that it is devoid of specific functional significance and instead is of more general [significance].”

(Cajal, 1899)

Thus, Cajal and other authors thought that it was essential to carry out comparative histological studies to see whether any structural peculiarities existed in the human cerebral cortex that might yield a key to specific human behaviors, a fundamental question in neuroscience which is still under debate (for reviews, see for example Rakic, 2009; DeFelipe, 2011; Sherwood et al., 2012; Kaas, 2013).

## FINAL CONSIDERATIONS

The process of ganglionic centralization was essential in the evolution of the nervous system. The discussion about the learning processes – which in a way in itself suggests the existence of some plasticity – indicates that the concept of plasticity became commonplace in academic circles in the 19th century, especially in the second half of this century when it became particularly widespread.

William James was one of the first scholars to propose that the nervous system – the organ system chiefly responsible for the processes of consciousness – is subject to changes, i.e., that it has plasticity. James, however, did not attempt to explain his idea at the anatomical level.

Cajal, who enthusiastically supports this hypothesis, constructed an argument firmly grounded in the conclusions stemming from his experimental results in favor of the neuronal theory. Based on such results, he attributes to the nervous system – in terms of evolution – the property of changing itself in response to the relationship between the organism and its environment. We note that Cajal was not the first to use the term plasticity to refer to the nervous system, but he was without doubt the first to attribute plasticity to connections between nerve cells and to explain them in adaptive terms in the process of ganglionic centralization, including the morphological differentiation of cell types.

## Conflict of Interest Statement

The authors declare that the research was conducted in the absence of any commercial or financial relationships that could be construed as a potential conflict of interest.
